# Post-radiation sciatic neuropathy: a case report and review of the literature

**DOI:** 10.1186/1477-7819-6-130

**Published:** 2008-12-11

**Authors:** Panagiotis D Gikas, Sammy A Hanna, Will Aston, Nicholas S Kalson, Roberto Tirabosco, Asif Saifuddin, Steve R Cannon

**Affiliations:** 1 Bone Tumour Unit, Royal National Orthopaedics Hospital, Stanmore, Middlesex, HA7 4LP, UK; 2 Oncology and Arthroplasty Fellow, Royal Prince Alfred Hospital, Camperdown, Sydney, Australia; 3 The Medical School, University of Manchester, Oxford Road, Manchester, M13 9PT, UK; 4 Department of Pathology, Royal National Orthopaedics Hospital, Stanmore, Middlesex, HA7 4LP, UK; 5 Department of Radiology, Royal National Orthopaedics Hospital, Stanmore, Middlesex, HA7 4LP, UK

## Abstract

**Background:**

Post-radiation peripheral neuropathy has been reported in brachial and cervical plexuses and the femoral nerve.

**Case presentation:**

We describe a patient who developed post-radiation sciatic neuropathy after approximately 3 years and discuss the pathophysiology, clinical course and treatment options available for the deleterious effects of radiation to peripheral nerves.

**Conclusion:**

This is the first case of post-radiation involvement of the sciatic nerve reported in the literature.

## Background

Post-radiation neuropathy was first reported in patients treated with radiotherapy to the axillary glands for malignant breast tumours. It has also been reported in patients treated for malignant lesions in the faciomaxillary region, where the cervical plexus, facial or hypoglossal nerves have been involved. Furthermore, two case reports exist in the literature of post-radiation femoral neuropathy [1,2]. To our knowledge, there has been no description so far of post-radiation involvement of the sciatic nerve.

In this article, we describe the case of a patient who developed post-radiation sciatic neuropathy after approximately 3 years and discuss the pathophysiology, clinical course and treatment options available for the deleterious effects of radiation to peripheral nerves.

## Case presentation

**Figure 1:**
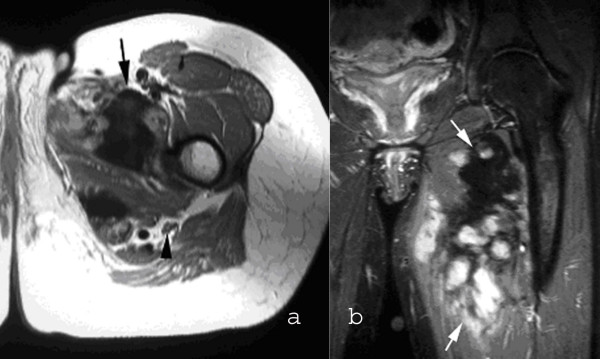
**MRI of the left thigh**. Axial T1W SE (a) and coronal STIR (b) images showing a poorly defined, lobular mass in the left adductor compartment (arrows) showing extensive areas of signal void due to fibrous tissue. Note the location of the sciatic nerve (arrowhead).

**Figure 2:**
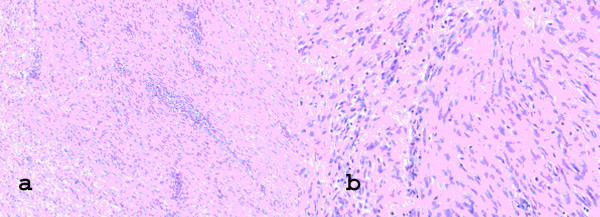
**Typical microsopic features of musculoaponeurotic fibromatosis**. Interlacing bundles of uniform spindle-shaped cells with pale oval nuclei and eosinophilic cytoplasm; there is a prominent collagen stroma.

A 22 year-old media student presented in 2001 with a two-year history of a mass in her left thigh adductor compartment. Magnetic resonance imaging (MRI) demonstrated a poorly defined, lobular mass in the left proximal adductor compartment, with significant areas of signal void consistent with the presence of excessive fibrous tissue (Figure 1). Needle biopsy confirmed a diagnosis of musculo-aponeurotic fibromatosis (Figure 2). The lesion was subsequently excised by complete adductor compartment resection with the exception of adductor longus. Post-operatively the patient completed a course of radiotherapy, receiving a total dose of 50 Gy in 25 fractions over five weeks.

Towards the end of 2002, she developed a swelling in the postero-medial thigh, distal to the previous irradiation field. MRI confirmed recurrence just above the level of the femoral condyles. In December 2002, she underwent further resection followed by a further course of radiotherapy (30 Gy in 15 fractions over 4 weeks) with an inch overlap with the previous radiation field superiorly.

In June 2003, the patient developed a further proximal thigh recurrence in the previously surgically treated area, within the initial radiotherapy field. She was started on Tamoxifen and further excision performed.

In March 2004, she started complaining of progressive weakness of dorsiflexion of her left foot, associated with pain around the medial aspect of the foot and sole. On clinical examination, she had normal hip flexion/extension and abduction with almost absent adduction, and normal knee flexion and extension. Foot flexion and inversion was 4/5. Foot and toe extension and eversion were 2/5. The ankle tendon reflex was absent. There was normal sensation on the anterior and posterior aspects of her thigh indicating that the posterior cutaneous nerve of the thigh coming from the sacral plexus was intact and hence that any lesion was distal to the sacral plexus. She had almost no sensation in the sole of her foot with reduced perception of touch on the dorsum of her foot. When palpating along the course of the sciatic nerve a rather dense region of local scarring was present on the posterior aspect of the thigh approximately 10 cm from the knee.

**Figure 3:**
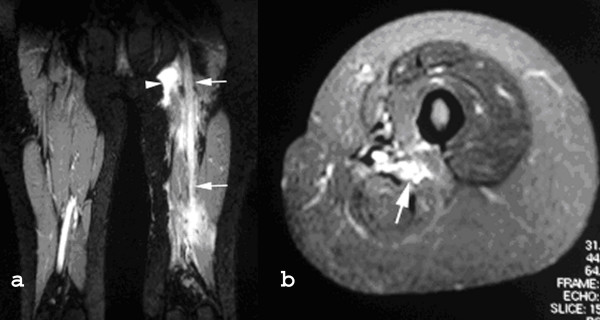
**Follow up MRI of the thigh**. Coronal STIR (a) and axial fat suppressed T2W FSE (b) images showing diffuse swelling and oedema of the sciatic nerve (arrows) and a postoperative seroma in the groin (arrowhead).

Electrophysiological assessment indicated a non localizing sciatic nerve sciatic nerve injury. Based on the clinical findings and investigations, a diagnosis of radiation-induced injury to the sciatic nerve was made, affecting the common peroneal portion more that the tibial portion. Repeat MRI of the left thigh demonstrated a seroma in the left groin and diffuse oedema and swelling of the left sciatic nerve (Figure 3). A decision to perform neurolysis of the sciatic nerve was made, with a view to freeing the nerve from any associated scar tissue, thereby halting any further deterioration in function.

At the most recent follow up in 2008, the patient is free from recurrence. There has been no further deterioration in sciatic nerve function but weakness of foot dorsiflexion persists, necessitating use of a splint.

## Discussion

### Pathophysiology and clinical course

Very little is known about the pathophysiology and the histopathological changes that occur in peripheral nerves after therapeutic irradiation. Early experimental studies indicated that the peripheral nerves are extremely radioresistant. However, the follow up time was short and it is likely that the injury did not have an opportunity to develop [3].

Today we know that post-irradiation neuropathy occurs both directly and indirectly: directly by the harmful effect of the radiation on the nerve itself, and indirectly by the fibrosis that radiation causes in the tissue around the nerve [2].

Direct effects of irradiation on nerve include bioelectrical alterations (subnormal action potentials, altered conduction time), enzyme changes, abnormal microtubule assembly, altered vascular permeability and neurilemmal damage. All of these changes are observed experimentally within 2 days after irradiation and are all dose dependent and irreversible [4-6].

The secondary damage to the nerve is due to the extensive fibrosis of the connective tissue around the nerve, which becomes densely hyalinised. There is also a progressive loss of elasticity and the development of contractures that ultimately consolidate the adjacent structures with the nerve. In addition, the decreased vascularity of the area may destroy some adjacent peripheral nerves. Regeneration of the affected nerves may be impeded [2]. In a report of findings at autopsy in two patients who had post-irradiation brachial-plexus syndrome [7], varying degrees of fibrosis of the neurilemma, as well as demyelinization and fibrous replacement of the fibrils, were described. Mendes et al. in histological examination of femoral nerve branches removed during surgical decompression of the femoral nerve, in a patient with post-irradiation femoral neuropathy, also found demyelinated nerve fibres surrounded by abundant scar tissue with areas of hyalinization [2].

Peripheral nerve damage is a rare but understandably major complication of radiation therapy associated with significant morbidity. The frequency of injury reported from some of the older studies is probably higher than would occur today as prior to the advent of CT and MRI, larger fields were used because of greater uncertainty about the dimensions of the tumour.

In studies looking into post-irradiation neuropathy involving the brachial and cervical plexuses after radiotherapy for breast carcinoma it was found that symptoms generally begin within one to two years after treatment and are initially mainly sensory (e.g. burning pain, numbness, paresthesia) [7,8]. Any motor deficits that develop are usually delayed for about eighteen months and include paresis of a group of muscles and complete paralysis of the arm [9]. Stoll et al. and Powell et al. have both found a direct relationship between the dosage of radiation and the severity/time of appearance of symptoms [7,10].

In a review of radiation injury to peripheral nerves published by Giese and Kinsella the authors conclude that peripheral neuropathy is relatively infrequent at lower doses per fraction [11]. They expressed concern that co-factors such as radiosensitizers, chemotherapeutic agents and surgical manipulations could possibly increase the incidence. Breast cancer patients receiving cytotoxic chemotherapy had a higher incidence of radiation induced brachial plexopathy compared to those having radiation only following mastectomy [12].

Any peripheral nerve may be affected by post-radiation neuropathy and it is likely that the unique location of this tumour reflects 1) the special site of radiation therapy and 2) the repeated doses of radiation administered [12].

Latency is an important factor to be considered when evaluating nerve injury [12]. Stoll and Andrews did not observe any neuropathy occurring before 5 months, with a majority occurring between 10 and 22 months after irradiation. They also noted that the higher dose group did show signs earlier than the lower dose group. Powell et al. did not observe any nerve injury prior to 10 months post-irradiation, whereas reports exist in the literature of neuropathies occurring as late as 11 years after irradiation for breast cancer. Therefore, latency, as our case demonstrates, is a very important factor to be considered, since short follow-up times may underestimate the true incidence of post-irradiation injury to peripheral nerves.

### Management

When considering management of post-irradiation peripheral neuropathy, it is important to realise that an unalterable condition is the status of the patient's underlying malignancy prior to initiation of treatment, including tumour size, location and structures involved/destroyed [12]. Furthermore, release of entrapped nerves from a fibrous mass can be challenging even for the most skilled surgeon. Therefore, a short life expectancy coupled with uncertainty of recovery from surgical intervention make conservative management more appropriate.

Also important in overall response to and recovery from therapy is the general health of the patient and, if a child, the stage of development and growth [12]. If surgery is a part of the overall treatment, as was in our case, then the extent of the surgical resection and the techniques used are also of major importance to post-therapy function. In addition, the long-term soft tissue response to radiation is a complex function of many radiation related factors (total dose, dose volume and distribution, fraction size, dose rate, treatment interval and overall treatment time) some of which are poorly understood. Important non-radiation factors that play a role in influencing the development, progression and response to treatment of post-radiation neuropathy, include other therapies such as surgery and chemotherapy, major organ system performance, overall activity level and chronic conditions such as hypertension, diabetes and connective tissue disorders.

In patients who have a good life expectancy after tumour excision, an increasing motor deficit and/or intolerable pain in the distribution of a peripheral nerve may present some years after the initial treatment. Some patients may require surgical release of the radiation induced scar tissue surrounding the nerve. However, the patient has to be aware of the uncertainty of recovery.

When a decision has been made to pursue a surgical path, treatment should not be delayed as research has shown that pathological changes in a peripheral nerve restricted by fibrosis are progressive.

## Conclusion

Despite being a rare entity, post-radiation peripheral neuropathy can be associated with significant morbidity. Further research is crucial in identifying the major pathophysiological mechanisms, both direct and indirect, underlying damage to peripheral nerves following therapeutic radiation. A good understanding of pathophysiology at a cellular/molecular level is essential for the development, in the future, of appropriate prophylactic measures for people requiring radiotherapy.

## Consent

Written informed consent was obtained from the patient for publication of this case report and any accompanying images. A copy of the written consent is available for review by the Editor-in-Chief of this journal.

## Competing interests

The authors declare that they have no competing interests.

## Authors' contributions

PG, WA, SH, and NSK reviewed the literature, wrote the Background and Case presentation sections, the Conclusion and edited the manuscript. RT described the histological findings and confirmed and edited the manuscript. AS described the radiological findings and confirmed and edited the manuscript. SRC conceived the case report and helped draft the manuscript.
